# The phosphokinase activity of IRE1ɑ prevents the oxidative stress injury through miR‐25/Nox4 pathway after ICH

**DOI:** 10.1111/cns.14537

**Published:** 2023-11-23

**Authors:** Yuhui Liao, Juan Huang, Zhenhua Wang, Zhengyu Yang, Yue Shu, Shengwei Gan, Zhixu Wang, Weitian Lu

**Affiliations:** ^1^ Department of Anatomy, Basic Medical College Chongqing Medical University Chongqing China; ^2^ Institute of Neuroscience, Basic Medical College Chongqing Medical University Chongqing China; ^3^ Medical College Sichuan University of Arts and Science Dazhou China

**Keywords:** intracerebral hemorrhage, IRE1α, miR‐25‐3p, Nox4, oxidative stress

## Abstract

**Background:**

Endoplasmic reticulum (ER) stress and oxidative stress are the major pathologies encountered after intracerebral hemorrhage (ICH). Inositol‐requiring enzyme‐1 alpha (IRE1α) is the most evolutionarily conserved ER stress sensor, which plays a role in monitoring and responding to the accumulation of unfolded/misfolded proteins in the ER lumen. Recent studies have shown that ER stress is profoundly related to oxidative stress in physiological or pathological conditions. The purpose of this study was to investigate the role of IRE1α in oxidative stress and the potential mechanism.

**Methods:**

A mouse model of ICH was established by autologous blood injection. The IRE1α phosphokinase inhibitor KIRA6 was administrated intranasally at 1 h after ICH, antagomiR‐25 and agomiR‐25 were injected intraventricularly at 24 h before ICH. Western blot analysis, RT‐qPCR, immunofluorescence staining, hematoma volume, neurobehavioral tests, dihydroethidium (DHE) staining, H_2_O_2_ content, brain water content, body weight, Hematoxylin and Eosin (HE) staining, Nissl staining, Morris Water Maze (MWM) and Elevated Plus Maze (EPM) were performed.

**Results:**

Endogenous phosphorylated IRE1α (p‐IRE1α), miR‐25‐3p, and Nox4 were increased in the ICH model. Administration of KIRA6 downregulated miR‐25‐3p expression, upregulated Nox4 expression, promoted the level of oxidative stress, increased hematoma volume, exacerbated brain edema and neurological deficits, reduced body weight, aggravated spatial learning and memory deficits, and increased anxiety levels. Then antagomiR‐25 further upregulated the expression of Nox4, promoted the level of oxidative stress, increased hematoma volume, exacerbated brain edema and neurological deficits, whereas agomiR‐25 reversed the effects promoted by KIRA6.

**Conclusion:**

The IRE1α phosphokinase activity is involved in the oxidative stress response through miR‐25/Nox4 pathway in the mouse ICH brain.

## Graphical abstract

The proposed pathway by which IRE1α regulates the oxidative stress response via miR‐25‐3p/Nox4 after ICH. ① ICH induces ER stress and activates the phosphokinase activity of IRE1α. ② Self‐phosphorylated IRE1ɑ increases the expression of miR‐25‐3p. ③ The complementary combination of miR‐25‐3p with Nox4 mRNA inhibits the translation of Nox4 mRNA and subsequently downregulated expression of Nox4, which leads to ROS production decrease, especially H_2_O_2_

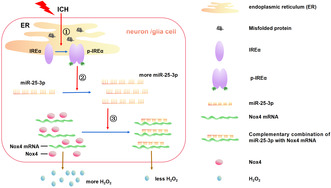

## 1 INTRODUCTION

Intracerebral hemorrhage (ICH) is a subtype of stroke, which accounts for 10%–15% of all strokes characterized by high mortality and morbidity.^1^, ^2^ A series of pathophysiological processes, such as brain edema, blood–brain barrier disruption, neuroinflammation, mitochondrial dysfunction, neuronal apoptosis, etc., are triggered by the initial mass mechanical effect of intracerebral hematoma and intrahematoma hemolytic products in ICH brain.^3^, ^4^ Recently, myriad studies started to focus on the role of various stress events in adaptive response and adverse reactions in neurological diseases. It has been established that endoplasmic reticulum stress (ER stress) and oxidative stress are involved in the complicated pathophysiological processes after ICH insult.^5^, ^6^ However, the complicated interaction between the two stress responses in ICH brain remains to be revealed.

The endoplasmic reticulum (ER) is a multifunctional organelle and is mainly responsible for proteins synthesis and folding as well as calcium storage. Some unbalanced factors, such as ischemia, hypoxia, and pH changes, will disturb the homeostasis of the ER and lead to unfolded or misfolded proteins accumulating in the ER lumen, resulting in ER stress.^7^, ^8^ In adaptive ER stress conditions, the unfolded protein response (UPR), a set of tightly controlled regulatory programs, is activated to reconstruct the protein‐folding homeostasis and to orchestrate the recuperation of ER function.^7^ However, under overwhelming or prolonged ER stress, UPR fails to restore the normal function of ER, and the apoptotic cascade will be induced in cells.^9^ UPR is activated through three ER transmembrane sensors, protein kinase RNA (PKR)‐like ER kinase (PERK), activating transcription factor 6 (ATF6), and inositol‐requiring protein 1 (IRE1).^10^, ^11^ Among them, IRE1 is the most conserved and the oldest branch one with two isoforms, IRE1α and IRE1β. As an endoplasmic reticulum transmembrane protein, IRE1α is expressed ubiquitously with the function of both a kinase and a ribonuclease (RNase). The mRNA encoding the transcription for X‐box binding protein‐1 (XBP1) could be cleaved by ribonuclease activity, resulting in the production of spliced XBP1 (XBP1s). XBP1s improves protein‐folding capacity and misfolded protein degradation in the ER lumen.^12^ In addition to initiating the ER stress survival pathway, sustained activation of IRE1α can induce downstream apoptotic signaling molecules like caspase‐2 and CHOP/GADD153 to cause cell apoptosis.^13^, ^14^ For this reason, the IRE1α activity is a key crossroad for the UPR signaling pathway switching from an adaptive response to negative consequences.

In addition to those coding RNAs such as XBP1 mRNA, the IRE1α RNase can also lead to the cleavage of a variety of noncoding RNAs including miRNAs.^15^ MicroRNAs (miRNAs) are a group of small single‐stranded noncoding RNAs (19–24 nucleotides) capable of regulating gene expression by binding to the 3′ untranslated region (UTR) of the mRNAs.^16^, ^17^, ^18^ It was found that miR‐25‐3p is involved in many pathological and physiological processes of the nervous system, including regulating neuroinflammation, neural stem cell proliferation, etc.^19^, ^20^ Recently, the role of miR‐25‐3p in regulating oxidative stress has attracted widespread attention from researchers. For example, miR‐25‐3p took antioxidant effects on primarily cultured neurons through targeting OXSR1,^21^ and cardiomyocytes could be protected by miR‐25‐3p against oxidative damage via downregulating mitochondrial calcium uniporter.^22^

Oxidative stress is a series of responses to reactive oxygen species (ROS) increasing in the body caused by the excessive ROS generation or the weaken of ROS scavenging system. Nicotinamide adenine dinucleotide phosphate oxidase 4 (Nox4) is a subtype of the Nox family, which is one of the main sources of ROS in mammals.^23^ Our searching results in the miRNA target prediction software showed that miR‐25‐3p contains nucleotide sequences complementary to Nox4 mRNA 3’‐UTR sequences (see Results section), which indicates that miR‐25‐3p is likely a regulator of Nox4 expression. At the same time, some studies confirmed that miR‐25‐3p is involved in the regulation of Nox4 expression in hydroquinone‐treated leukemia cells and diabetic peripheral neuropathy.^24^, ^25^ In view of the above reasons, the role of miR‐25‐3p on regulating oxidative stress may be realized via Nox4 in ICH brain, which is waiting for being revealed.

Given that a large body of miRNAs are cleaved by activated IRE1α, miR‐25‐3p is likely one of the cleavage substracts of IRE1α RNase activity. Considering the role of miR‐25‐3p on oxidative stress, miR‐25‐3p is probably an important factor connecting with IRE1α activation during ER stress and Nox4 activation during oxidative stress following ICH brain injury. We will explore whether could IRE1α activation induce oxidative stress via miR‐25‐3p/Nox4 pathway after ICH insult in the present study.

## 2 MATERIALS AND METHODS

### 2.1 Experimental animals

A total of 287 Kunming mice (8–10 weeks, weight 25–30 g) were purchased from the Animal Center of Chongqing Medical University. Among them, 15 mice were excluded due to death during surgery. Table 1 displays the utilization of our different mouse groups and the mortality rate among the mice. All mice were provided with standard rodent food and water and accommodated in an air‐conditioned environment with a 12 h light/dark cycle. All procedures were approved by the Animal Ethics Committee of Chongqing Medical University.

**TABLE 1:** The mortality rate of mice.

Groups	WB	PCR	BWC	IF staining	HE/Nissl staining	Hematoma volume	DHE staining	H_2_O_2_ contents	MWM/EPM/body weight test	Mortality	Total
Sham	8	8	4		share with IF staining (4)	4	4	4	8		44
ICH (6 h, 12 h, 1d, 3d, 7d)	20	20		4 (ICH‐12 h)						4 (8%)	48
ICH + vehicle	4	4	4		4	4	4	4	8	2 (5%)	38
ICH + KIRA6(5 mg/kg)	4	4	4		4	4	4	4	8	2 (5%)	38
ICH + KIRA6(10 mg/kg)	4					4					8
ICH + KIRA6(20 mg/kg)	4					4				1 (11%)	9
ICH + KIRA6 + antagomiR‐NC	4	4	4			4	4	4		1 (4%)	25
ICH + KIRA6 + antagomiR‐25	4	4	4			4	4	4		3 (11%)	27
ICH + KIRA6 + agomiR‐NC	4	4	4			4	4	4		1 (4%)	25
ICH + KIRA6 + agomiR‐25	4	4	4			4	4	4		1 (4%)	25
Total	60	52	28	8	8	36	28	28	24	15 (5%)	287

### 2.2 Experimental design

**FIGURE 1:**
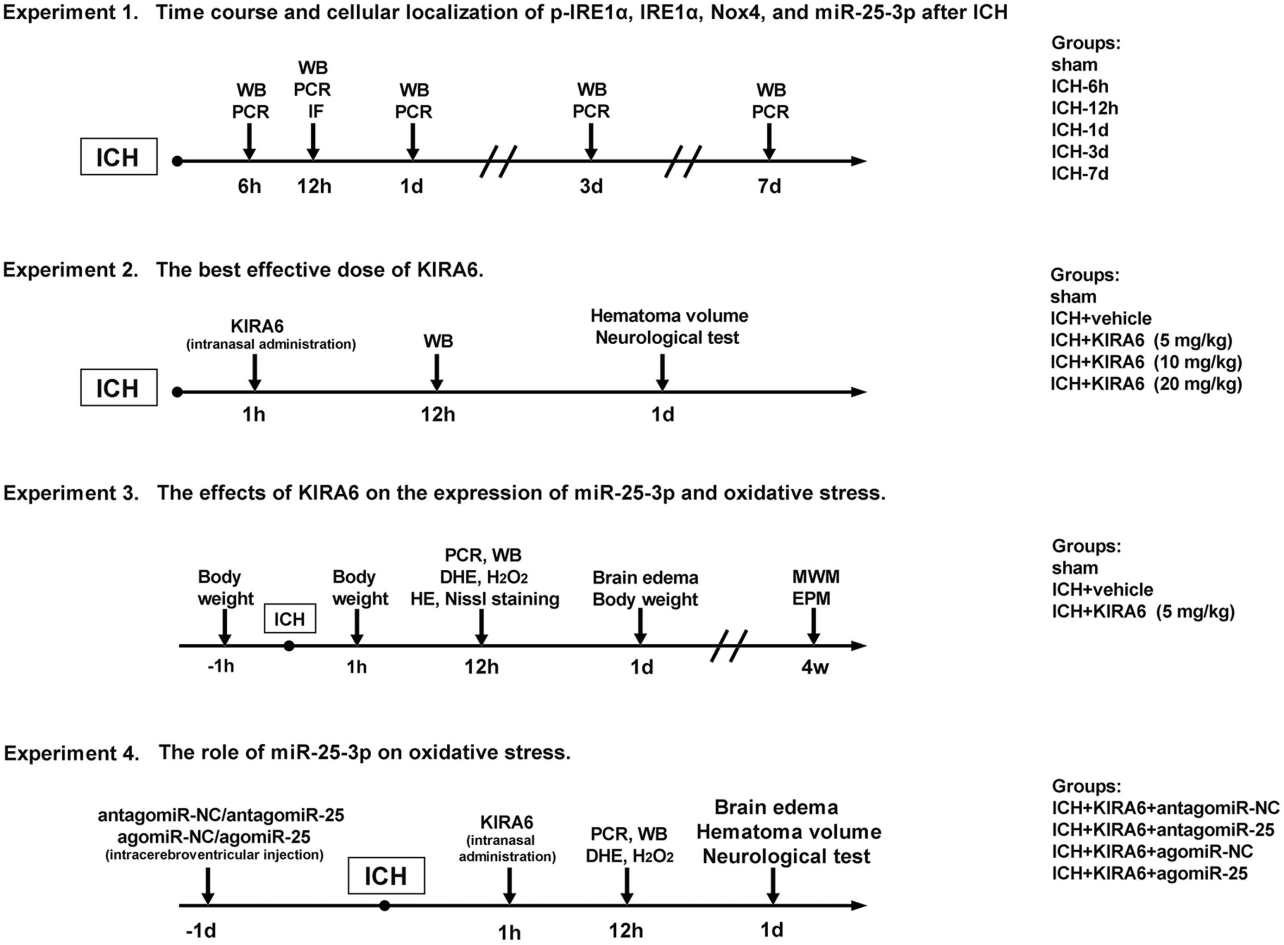
Four‐part experimental design.

Four separate experiments were designed in the following way (Figure 1).

#### 2.2.1 Experiment 1

In order to determine the time course of endogenous IRE1α, p‐IRE1α, miR‐25‐3p, and Nox4 expression levels in the right cerebral hemisphere after ICH, reverse transcription quantitative real‐time polymerase chain reaction (RT‐qPCR) and western blot analysis were performed. According to different time points after ICH, the mice were distributed randomly and equally into six groups: the sham group and the ICH groups (6 h, 12 h, 1 d, 3 d, 7 d). Furthermore, immunofluorescence double staining was used to determine the cellular localization of p‐IRE1α, respectively, with the marker of astrocytes (glial fibrillary acidic protein, GFAP), the marker of neurons (neuronal specific nuclear protein, NeuN) and the marker of microglia (ionized calcium‐binding adaptor molecule 1, Iba‐1). The mice were euthanized at 12 h post‐ICH and brain samples were collected for immunofluorescence staining.

#### 2.2.2 Experiment 2

In order to determine the best effective dose of phosphokinase‐inhibiting RNase attenuator 6 (KIRA6, an allosteric IRE1α inhibitor), the mice were distributed randomly into five groups: sham, ICH + vehicle, ICH + KIRA6 (5 mg/kg, low dose), ICH + KIRA6 (10 mg/kg, medium dose), and ICH + KIRA6 (20 mg/kg, high dose). Hematoma volume and neurological tests were detected at 1 day post‐ICH. Western blot analysis was performed at 12 h after ICH.

#### 2.2.3 Experiment 3

In order to determine the effects of KIRA6 on the level of miR‐25‐3p, Nox4, and Nrf2 expression, oxidative stress, neurobehavior function, brain edema, body weight after ICH, mice were distributed randomly into three groups: sham, ICH + vehicle, ICH + KIRA6 (5 mg/kg). RT‐qPCR, Western blot, dihydroethidium (DHE) staining, Hematoxylin and Eosin (HE) staining, Nissl staining, and hydrogen peroxide (H_2_O_2_) content analysis were performed at 12 h after ICH. Brain water content (BWC) and body weight were estimated at 1 day post‐ICH. Morris Water Maze (MWM) and Elevated Plus Maze (EPM) were performed at 4 weeks after ICH.

#### 2.2.4 Experiment 4

In order to determine whether miR‐25‐3p is involved in the relationship between IRE1α and oxidative stress, the antagomiR‐NC/antagomiR‐25 or agomiR‐NC/agomiR‐25 were used in this study. The mice were distributed randomly into four groups: ICH + KIRA6 + antagomir‐NC, ICH + KIRA6 + antagomiR‐25, ICH + KIRA6 + agomir‐NC, ICH + KIRA6 + agomiR‐25. AntagomiR‐NC, antagomiR‐25, agomiR‐NC, and agomiR‐25 were injected at 24 h before ICH. Neurobehavioral function, hematoma volume, and BWC were estimated at 1 day post‐ICH. RT‐qPCR, Western blot, DHE, and H_2_O_2_ content analysis were analyzed at 12 h post‐ICH.

### 2.3 ICH model establishment

The ICH model was established through autologous blood infusion based on earlier studies.^26^, ^27^, ^28^ The mice were anesthetized and secured in a stereotactic apparatus. A longitudinal incision was made to expose the skull. The stereoscopic coordinates were 2.3 mm lateral and 0.2 mm anterior to the bregma. A total of 30 μL autologous blood drawn from the tail was injected into the hole by a microinjection syringe. The blood injection rate during the whole injection process was 2 μL/min. After injection, the needle was retained for 10 min and slowly withdrawn at a rate of 1 mm/min. The tiny hole was then plugged with bone wax and the wound is stitched with sutures. For sham groups, the operation procedures were performed with needle insertion only. After surgery, rectal temperature was maintained at 37.0 ± 0.5°C through the use of a temperature‐controlled heating pad until the mice regained consciousness. Subsequently, the mice were housed in a temperature‐controlled environment at a constant room temperature of 22°C. Mice body weight were monitored at three time points: 1 hour pre‐ICH, 1 hour post‐ICH, and 1 day post‐ICH.

### 2.4 Intranasal administration

KIRA6 was dissolved in dimethyl sulfoxide (DMSO) as the stock solution. After being anesthetized, the mice were placed in a supine position. A total volume of 5 μL KIRA6 (diluted to the final dose) or vehicle was divided into four drops and applied into both sides of the nostril alternatively. Each side nostril was administered 1.25 μL KIRA6 or vehicle each time with 2 min intervals.

### 2.5 Intracerebroventricular injection

To investigate the effects of miR‐25‐3p on oxidative stress after ICH, antagomiR‐NC/antagomiR‐25 (0.5 nmol/mice, B05002, GenePharma) or agomiR‐NC/agomiR‐25 (0.5 nmol/mice, B06002, GenePharma) were administrated into the lateral ventricle at 24 h before ICH. Briefly, a tiny hole was made in the parietal region (1.7 mm deep, 1.5 mm posterior, and 1.5 mm lateral) as previously described,^28^ and the antagomiR‐NC/antagomiR‐25 or agomiR‐NC/agomiR‐25 (10 μL) solution was injected into the lateral ventricle at a flow rate of 2 μL/min. After injection, the needle was retained for 10 min and slowly withdrawn at a rate of 1 mm/min. The tiny hole was then plugged with bone wax and the wound is stitched with sutures.

### 2.6 Neurological score assessment

After the establishment of the mouse model a series of behavioral tests, including the composite Garcia, corner turn test, MWM, and EPM were performed. The composite Garcia test and the corner turn test were used to assess neurological function at 1 day post‐ICH. In the assessment process, we adhere to the principle of blind evaluation. The composite Garcia test consists of seven elements, which are spontaneous activity, climbing, vibrissae proprioception, lateral turning, symmetry of limb movement, forelimb walking, and axial sensation. In the corner turn test, mice were placed in a 30° corner with their heads toward the corner and were allowed to take either a right or left turn to exit the corner, the right or left turn times were recorded. These trials were performed 10 times per day, with intervals of at least 30 s between each test. The number of (right turns/all turns 100%) was used to calculate the score.^29^, ^30^

After the establishment of the mouse model for 4 weeks, the MWM was used to test the spatial learning and memory abilities of rodents.^31^ Prior to the commencement of the tests, the mice are acclimated in a habituation room to familiarize themselves with the environment for 1 h. It consists of a circular container with a diameter of 120 cm and a depth of 50 cm. The container is filled with nontoxic brown dye‐stained circulating water at a constant temperature, maintained at room temperature (20 ± 1°C). The maze is designated with two imaginary axes, where each line divides the maze into four equal quadrants vertically to the other line. The endpoints of each line divide the maze into four reference points: east, south, west, and north. A platform is located in the center of one of the quadrants, submerged 0.5 cm below the water surface. Each mouse is gently released into the maze from the expected starting position, facing away from the container walls. To promote the development of spatial memory, the starting point is different each day. After a 24 h open field test, each mouse undergoes four consecutive training trials per day, with a 20 min interval, lasting for 5 days, in order to locate the hidden platform. If a mouse reaches the hidden platform and stays there for at least 2 s, or if it fails to find the platform within 60 s, the training trial ends. Mice that fail to find the platform within the designated time are guided to the platform and allowed to stay there for 10 s. After the session, the mice are removed, dried, and returned to their home cages. Within 24 h after the last training trial, the platform is moved for a probe test. The mice are released in a new position to ensure the use of spatial memory rather than a specific swimming path, and they are allowed to freely swim for 60 s. The time spent in the target quadrant and the number of crossings over the platform are recorded as variables for learning and memory abilities. The escape latency and first entry into the target (platform location area), target crossings, swim speed (cm/s), and time spent in the target quadrant during the probe test are recorded using an automated tracking system during the initial training.

After the establishment of the mouse model for 4 weeks, the EPM test was commonly used to assess the locomotor activity, exploration, and anxiety‐like behavior in rodents.^32^ Prior to the commencement of the tests, the mice are acclimated in a habituation room to familiarize themselves with the environment for 1 h. The EPM is made of opaque blue plastic material and consists of two open arms (30 × 6 cm) opposite to each other and perpendicular to two enclosed arms (30 × 6 cm), elevated 30 cm above the floor. Each mouse was placed near the center area, avoiding visibility of the enclosed arms, and allowed to freely explore for 5 min. A video tracking system automatically recorded the mouse's movement speed and percentage of time spent on the open and enclosed arms, as well as the number of entries into the center area during the 5 min session. Prior to each test, the subjects were cleaned with 75% alcohol to remove any olfactory cues.

### 2.7 Western blot analysis

After the mice were anesthetized and transcardially perfused with phosphate‐buffered saline (PBS), the right cerebral hemispheres of each group were removed. The protein samples were extracted in RIPA lysis buffer (P0013B, Beyotime) containing PMSF and then centrifuged at 12,000*g* for 30 min. The supernatants were mixed with loading buffer (P0015L, Beyotime) and boiled at 95°C. The proteins were electrophoresed on a 10% gel, transferred to PVDF membranes at 250 mA current and blocked at 37 °C for 2 h. The primary antibodies were anti‐Nox4 (A3656, 1:500, Abclonal), anti‐IRE1α (NB100‐2324, 1:500, Novus), anti‐pIRE1α (AF7150, 1:500, Affinity), anti‐Nrf2 (A21176, 1:500, Abclonal) and anti‐β‐actin (60008‐1‐Ig, 1:5000, proteintech). The membrane was then incubated with horseradish peroxidase (HRP)‐conjugated secondary antibodies (1:10000, Earthox) for 1 h. Immunoblots were performed using ECL kits (4AW011, 4A Biotech) and analyzed by Image J software (ImageJ, Germany).

### 2.8 Reverse transcription quantitative real‐time polymerase chain reaction (RT‐qPCR)

Total mRNA and miRNAs were extracted from the right cerebral hemisphere using the RNAiso Plus reagent (TaKaRa, Japan). RT‐qPCR detection was performed using the PrimeScriptTM RT Reagent kit and the Green® Premix Ex TaqTM II (Tli RNaseH Plus) kit (Takara, Japan). Ultimately, miR‐25‐3p and Nox4 mRNA expression were calculated by the 2^−ΔΔct^ method. The sequence of RT‐PCR primers is listed in Table 2.

**TABLE 2:** The sequence of primers used in RT‐qPCR.

Names		Sequences
mmu‐miR‐25‐3p	Forward	GCGCATTGCACTTGTCTCG
	Reverse	AGTGCAGGGTCCGAGGTATT
U6	Forward	GGAACGATACAGAGAAGATTAGC
	Reverse	AGTGCAGGGTCCGAGGTATT
Nox4	Forward	CCCTCCTGGCTGCATTAGTC
	Reverse	CGGTAAAGTCTCTCCGCACA
GAPDH	Forward	CAGTGGCAAAGTGGAGATTGTTG
	Reverse	TCGCTCCTGGAAGATGGTGAT

### 2.9 Preparation of frozen sections

As previously described,^33^ the brain samples were perfused with 4% paraformaldehyde and then dehydrated in gradients of 10%, 20%, and 30% sucrose solution. The samples were divided into 10 μm thick coronal sections, and then we collected consecutive sections within a range of 200 μm both anterior and posterior to the injection site. The well‐prepared frozen sections are stored in a −20°C refrigerator for immunofluorescent staining, HE staining, and Nissl staining.

### 2.10 Immunofluorescence staining

The prepared frozen sections were washed with PBS and incubated with 5% bovine serum albumin (BSA, AR0004, Boster). The sections were then incubated with the following primary antibodies: rabbit anti‐p‐IRE1α (1:50, AF7150, affinity), mouse anti‐GFAP (1:100, 3670S, Cell Signaling Technology), mouse anti‐NeuN (1:200, 94403S, Cell Signaling Technology), and mouse anti‐Iba‐1 (1:100, ab283319, Abcam). The sections were washed with PBS and incubated with the corresponding secondary antibodies (1:100, Earthox). Furthermore, the cell nuclei were stained with 4′,6‐diamidino‐2‐phenylindole (DAPI) Fluoromount‐G (0100–20, Southern Biotech), and the fluorescent pictures were taken under a fluorescent microscope (IX73, Olympus). The Integrated Density analysis function of Image J software was used to quantify the immunostaining intensity of p‐IRE1α (Image J, Germany).

### 2.11 DHE Staining

The DHE staining method was used for the detection of ROS content in brain tissue. DHE (10 μmol/L) was injected intraperitoneally into the ICH model for 30 min before the mice were sacrificed.^34^ The brains were then sliced and placed on glass slides. The sections were incubated with DHE (S0063, Beyotime) in a dark container at 37°C for 30 min. Following rinsing of the excess reagent and staining of the nuclei, the fluorescent pictures were taken under a fluorescence microscope. To quantify the immunostaining intensity, we utilized the Integrated Density analysis function of Image J software (ImageJ, Germany).

### 2.12 HE staining

The stored frozen sections were rewarmed for 5 min, washed with water, stained with hematoxylin for 5 min, fractionated for 10 s, and stained with eosin for 1 min. The sections were then rinsed in 75% ethanol, 85% ethanol, 95% ethanol, and anhydrous ethanol for 2–3 s, transparented in xylene, sealed with neutral gum, and observed under a microscope.

### 2.13 Nissl staining

The stored frozen sections were rewarmed and washed in water for 2–4 s. The sections were then stained with a 1% aqueous toluidine blue solution for 5 min, washed in water, and rinsed in 70% ethanol, 95% ethanol, and anhydrous ethanol, respectively, transparented in xylene, sealed with neutral gum, and observed under a microscope. And then, we employed the automated cell counting function of Image J software (Image J,Germany) to calculate the number of surviving neurons.

### 2.14 H_2_O_2_ content

The mice were deeply anesthetized and transcardially perfused with PBS. The right cerebral hemisphere was then removed and mixed with 0.9% NaCl at a ratio of 1: 9, followed by mechanical homogenization. The content of H_2_O_2_ was measured by H_2_O_2_ assay kit (KGT018, KeyGEN) according to the instructions of the manufacturers. The absorbance at 405 nm was detected with a spectrophotometer, and the H_2_O_2_ content was analyzed based on a standard curve.

### 2.15 Hematoma volume

According to the manufacturer's instructions, the free hemoglobin assay reagent (A071‐1‐1, Jiancheng) was added to the prepared tissue supernatants and then incubated at 37°C. The absorbance was measured at 510 nm using a spectrophotometer.

### 2.16 Brain water content

BWC was determined at 1 day after ICH based on the dry‐wet weight method as previously described. Briefly, the brain samples were collected and divided into five sections: ipsilateral and contralateral basal ganglia, ipsilateral and contralateral cortex, and cerebellum. Each section was weighed using an electronic balance and then dried at 95°C for 72 h. The percentage of brain water content was calculated as follows: [(wet weight–dry weight)/wet weight×100%].

### 2.17 Statistical analysis

Statistical analyses were performed using the GraphPad Prism software (GraphPad Software, USA). Data were presented as means ± SD. The Shapiro–Wilk test was assessed the normal distribution of data within each group. Homogeneity of variance was evaluated using F test for unpaired *t*‐test analysis between two groups. Homogeneity of variance was evaluated using Brown‐Forsythe test for one‐way ANOVA between three and more groups, followed by multiple comparisons using Tukey's post hoc test. *p* < 0.05 was regarded statistically significant.

## 3 RESULTS

### 3.1 Time course of p‐IRE1α, IRE1α, Nox4 and miR‐25‐3p expression after ICH

**FIGURE 2:**
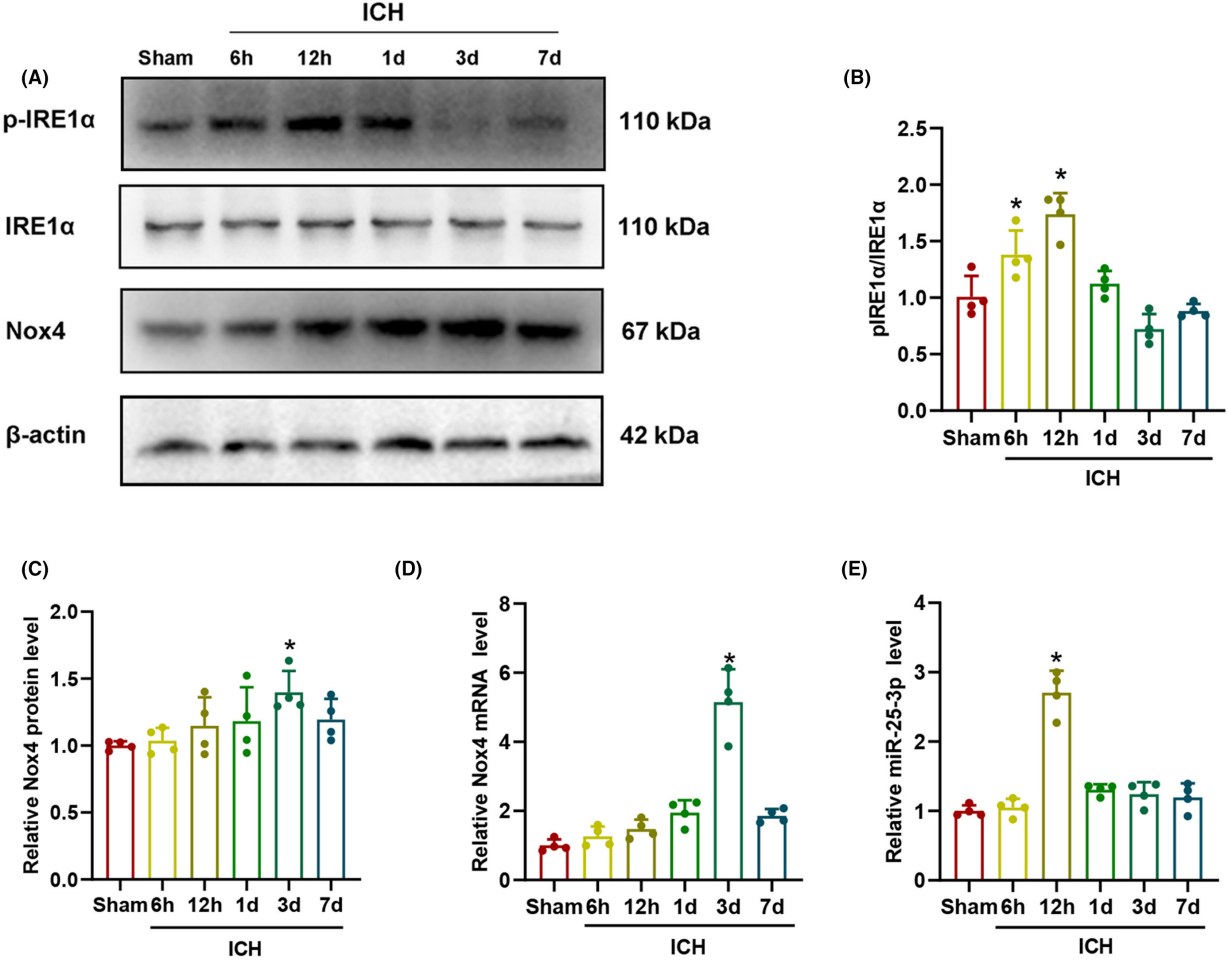
The expression levels of p‐IRE1α, IRE1α, Nox4, and miR‐25‐3p at different time points after ICH. (A) The protein bands of pIRE1α, IRE1α, and Nox4. (B,C) Quantification of the p‐IRE1α/IRE1α and Nox4 protein levels at various time points (*n* = 4). (D,E) Quantification of the Nox4 mRNA and miR‐25‐3p expression levels at various time points (*n* = 4). Data are the mean ± SD. *p < 0.05 vs. sham group.

The ratio of p‐IRE1α and IRE1α expression were measured by western blot at different time points after ICH. As shown in Figure 2A,B, the ratio of p‐IRE1α/IRE1α expression began to increase at 6 h after ICH, and peaked at 12 h compared with the sham group (*p* < 0.05). The expression levels of Nox4 protein and mRNA in ICH brain tissue were detected by western blot and RT‐qPCR. The results showed that the expression levels of Nox4 protein and mRNA began to increase at 6 h after ICH, and peaked at 3 d compared with the sham group (*p* < 0.05, Figure 2A,C,D). RT‐qPCR results showed that the expression level of miR‐25‐3p was markedly increased at 12 h after ICH compared with the sham group (*p* < 0.05, Figure 2E). These results demonstrated that the expression levels of p‐IRE1α and miR‐25‐3p were upregulated at the same time after ICH and earlier than that of Nox4.

### 3.2 p‐IRE1α was expressed in astrocytes, neurons, and microglia after ICH

**FIGURE 3:**
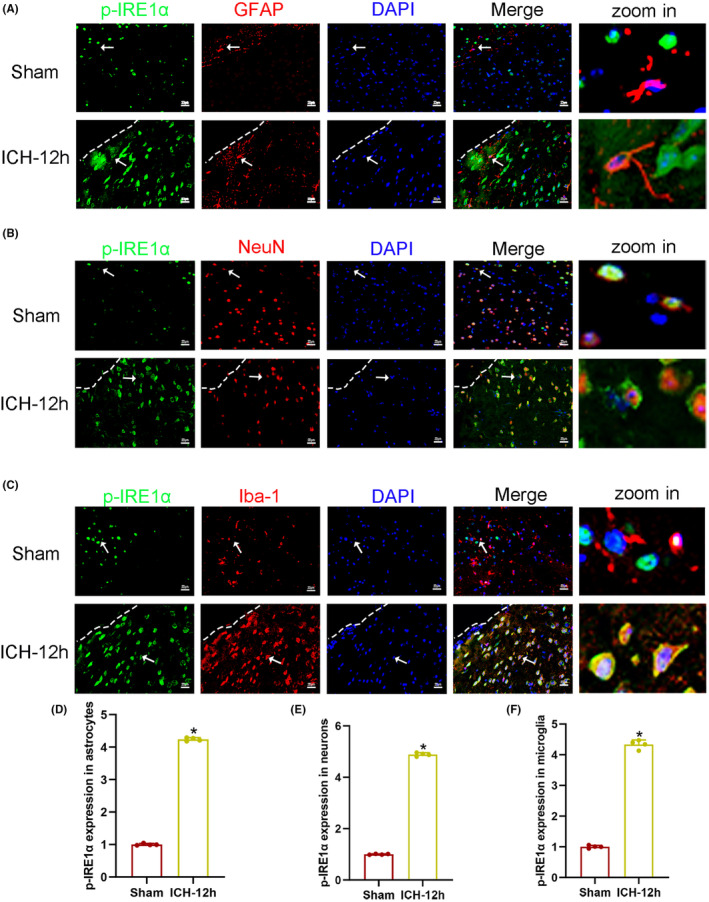
(A–C) Representative immunofluorescent images showing the co‐localization of p‐IRE1α with GFAP, NeuN, and Iba‐1, respectively, around the hematoma in sham and ICH‐12 h group (*n* = 4). The dotted line indicates the approximate boundary of the hematoma. The arrows point to co‐expression cells. The details are zoomed into the rightmost column. Scale bar = 20 μm. (D–F) Quantification of the intensity of p‐IRE1α (green fluorescence) in astrocytes/neurons/microglia in sham and ICH‐12 h group by Image J software (*n* = 4). Data are presented as mean ± SD. *p < 0.05 vs. sham group.

The localization of p‐IRE1α expression with GFAP, NeuN, and Iba‐1 were explored by double immunofluorescence staining in sham and ICH‐12 h group. As shown in Figure 3A,D, the fluorescence intensity of p‐IRE1α in astrocytes in ICH‐12 h group increased significantly compared with that in sham group (*p* < 0.05). As demonstrated in Figure 3B,E, the fluorescence intensity of p‐IRE1α increased in neurons in ICH‐12 h group compared with that in sham group (*p* < 0.05). As presented in Figure 3C,F, the fluorescence intensity of p‐IRE1α increased in microglia at 12 h after ICH (*p* < 0.05). These results indicated that p‐IRE1α was extensively expressed in astrocytes, neurons, and microglia, and its expression increased in various cell types after ICH.

### 3.3 KIRA6 dose‐dependently inhibited p‐IRE1α/IRE1α protein expression, increased hematoma volume and neurological function after ICH

**FIGURE 4:**
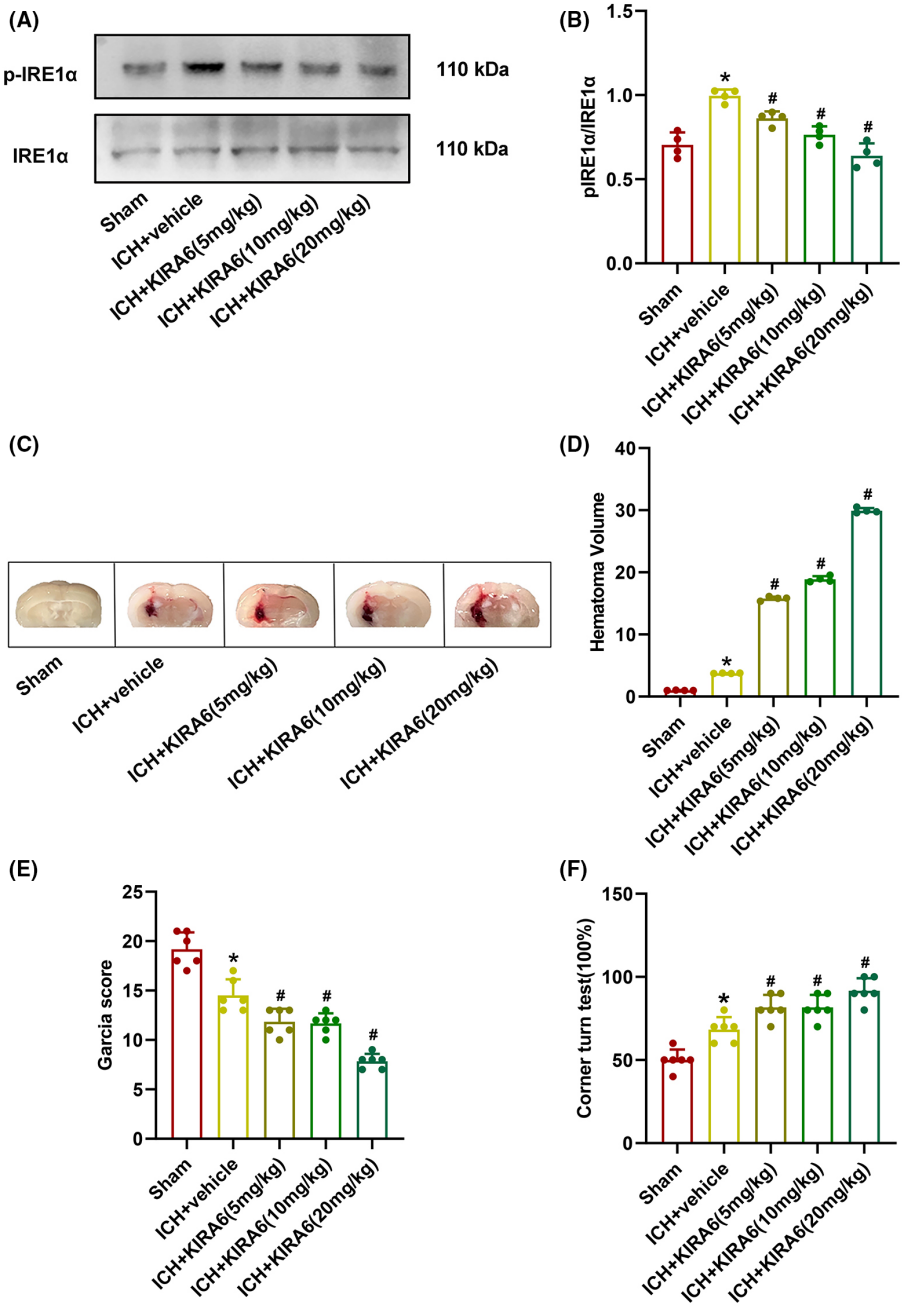
Effects of intranasal administration of KIRA6, respectively, at low dose (5 mg/kg), medium dose (10 mg/kg), and high dose (20 mg/kg) with p‐IRE1α/IRE1α protein expression, hematoma volume, and neurological function. (A) The protein bands of p‐IRE1α and IRE1α. (B) Quantification of the p‐IRE1α/IRE1α protein levels (*n* = 4). (C) Representative images showing the hematoma in the groups of sham, ICH + vehicle, and ICH + KIRA6 (5 mg/kg, 10 mg/kg, 20 mg/kg). (D) The analysis of hematoma volume in the groups of sham, ICH + vehicle, and ICH + KIRA6 (5 mg/kg, 10 mg/kg, 20 mg/kg). *n* = 4 per group. (E) The composite Garcia scores were evaluated in sham, ICH + vehicle, and ICH + KIRA6 (5 mg/kg, 10 mg/kg, 20 mg/kg). (F) The corner turn times were evaluated in sham, ICH + vehicle, and ICH + KIRA6 (5 mg/kg, 10 mg/kg, 20 mg/kg). *n* = 4 per group. Data are the mean ± SD. *p < 0.05 vs. sham group; ^#^ *p* < 0.05 vs. ICH + vehicle group.

The optimal effective dose of KIRA6 in the ICH mouse model was determined by western blot, hematoma volume assay, and neurobehavioral tests. The ratio of p‐IRE1α/IRE1α protein expression in the ICH + vehicle group was significantly higher than in the sham group by western blot. Three doses of KIRA6 (low 5 mg/kg, medium 10 mg/kg, high 20 mg/kg) could effectively downregulate the ratio of p‐IRE1α/IRE1α protein expression compared with the ICH + vehicle group, and the downregulation effect was KIRA6 dose‐dependent. (*p* < 0.05, Figure 4A,B).

The hematoma volume data revealed that the hematoma volume in the ICH + vehicle group were higher than that in the sham group. Three doses of KIRA6 could effectively increase the hematoma volume compared with the ICH + vehicle group, and the increasing effect was KIRA6 dose‐dependent (*p* < 0.05, Figure 4C,D).

The Garcia score decreased in the ICH + vehicle group compared with the sham group. KIRA6 could further decrease the score compared with the ICH + vehicle group, and the decreasing effect was dose‐dependent on KIRA6. (*p* < 0.05, Figure 4E).

The number of right turns increased after ICH compared with the sham group. KIRA6 could further increase the number compared with the ICH + vehicle group, and the increasing effect was dose‐dependent on KIRA6. (*p* < 0.05, Figure 4F).

Considering alleviating the additional distress to mice, the low dose (5 mg/kg) of KIRA6 was selected for further experiments in this study.

### 3.4 Administration of KIRA6 downregulated the expression level of miR‐25‐3p after ICH

**FIGURE 5:**
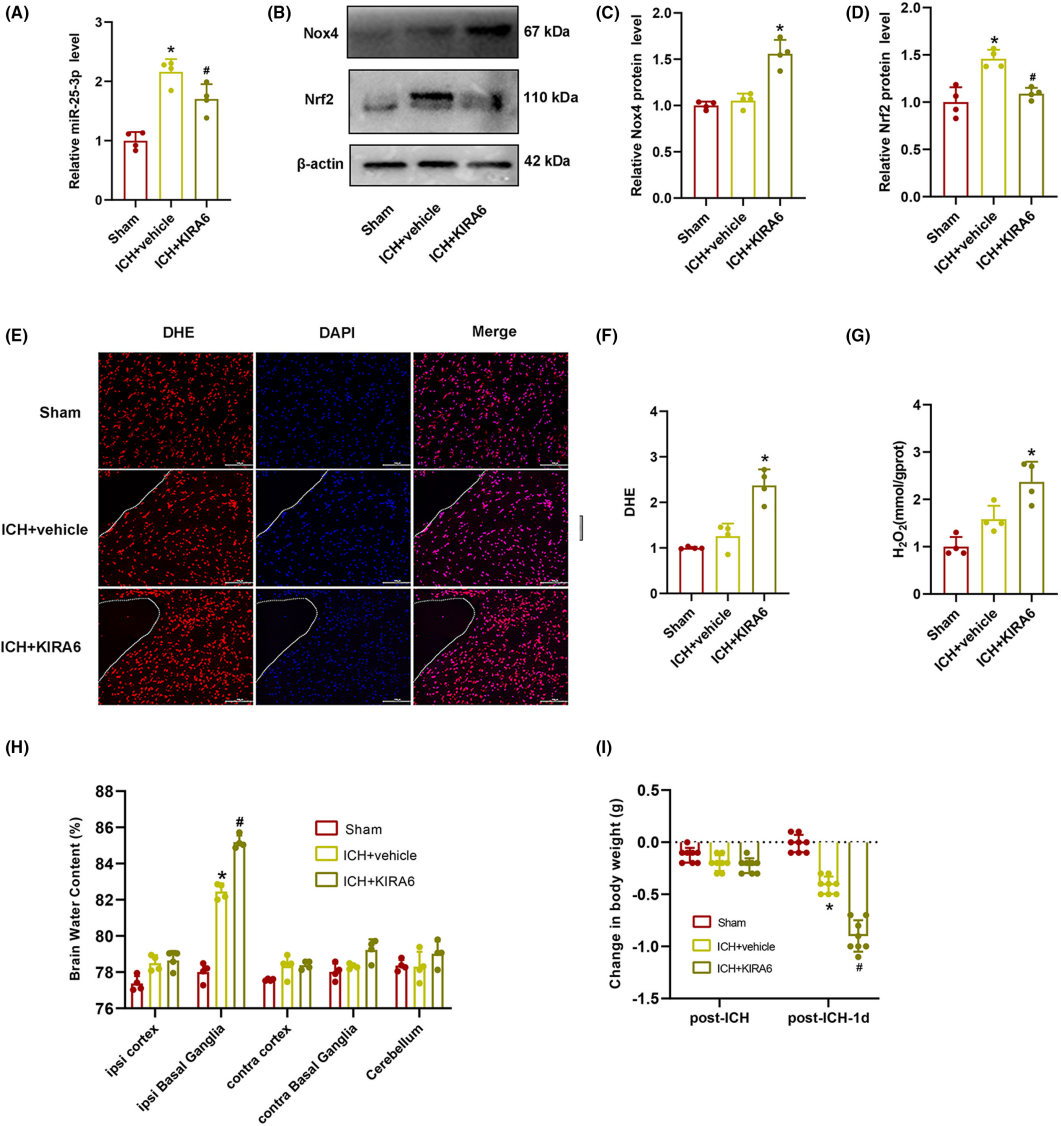
Effects of intranasal administration of KIRA6 on the expression levels of miR‐25‐3p and Nox4, oxidative stress, and brain edema. (A) RT‐qPCR analysis of miR‐25‐3p expression after KIRA6 administration (*n* = 4). Values are presented as mean ± SD. *p < 0.05 vs. sham group; ^#^ *p* < 0.05 vs. ICH + vehicle group. (B) Representative western blot bands of Nox4 and Nrf2. (C) Quantitative analysis of the relative expression levels of Nox4 (*n* = 4). *p < 0.05 vs. ICH + vehicle group. (D) Quantification of the Nrf2 protein levels (*n* = 4). *p < 0.05 vs. sham group; ^#^ *p* < 0.05 vs. ICH + vehicle group. (E) Representative DHE staining images around the hematoma. The boundary of the hematoma is indicated by the dotted line. Scale bar = 100 μm. (F) Quantification of the intensity of DHE (red fluorescence) by Image J software (*n* = 4). (G) The measurement of H_2_O_2_ contents (*n* = 4). Values are presented as mean ± SD. *p < 0.05 vs. ICH + vehicle group. (H) The analysis of brain edema in sham, ICH + vehicle, and ICH + KIRA6 groups (*n* = 4). (I) The bodies weight was evaluated in the groups of Sham, ICH + vehicle, and ICH + KIRA6 (*n* = 8). Data are the mean ± SD. *p < 0.05 vs. sham group; ^#^ *p* < 0.05 vs. ICH + vehicle group.

To verify the relation between IRE1α and miR‐25‐3p in the ICH mouse model, RT‐qPCR was performed following KIRA6 administration. The results demonstrated that miR‐25‐3p expression was increased after ICH compared with the sham group, whereas the expression level of miR‐25‐3p was suppressed by KIRA6 administration (*p* < 0.05, Figure 5A). Meanwhile, the results demonstrated that the IRE1α activity and miR‐25‐3p expression level changed toward the same direction after ICH and IRE1α inhibition, which indicated that miR‐25‐3p is not the cleavage substrate of IRE1α RNase. This conclusion is not consistent with our initial deduction that IRE1α activation may lead to the decay of miR‐25‐3p.

### 3.5 Administration of KIRA6 upregulated Nox4 protein expression, oxidative stress, brain injury, reduced body weight, exacerbated spatial learning and memory deficits, and increased anxiety levels after ICH

To determine whether the administration of KIRA6 has an effect on Nox4 and Nrf2 expression levels, western blot was performed. It was found that the expression level of Nox4 did not change markedly after ICH compared with the sham group, while administration of KIRA6 increased the level of Nox4 expression. Nrf2 is a key transcription factor in the biological antioxidant defense system. Under pathological conditions, Nrf2 binds to the antioxidant response element (ARE) to promote the transcription of antioxidant enzymes, including nicotinamide adenine dinucleotide phosphate (NADPH).^35^, ^36^, ^37^ The expression level of Nrf2 was higher after ICH compared with the sham group, while administration of KIRA6 suppressed the expression level of Nrf2. (*p* < 0.05, Figure 5B–D).

As one of the downstream products of Nox4, H_2_O_2_ has been found to play a crucial role in oxidative stress. DHE is one of the most commonly used fluorescent probes of superoxide anions. Therefore, DHE staining and H_2_O_2_ production were used to detect the oxidative stress in KIRA6‐administrated ICH mice. As shown in Figure 5E–G, the intensity of DHE fluorescence and H_2_O_2_ content was slightly increased in the ICH + vehicle group compared with the sham group, but the difference between the two groups was not statistically significant. KIRA6 dramatically enhanced the fluorescence intensity of DHE and H_2_O_2_ contents compared with the ICH + vehicle group (*p* < 0.05).

Brain water content was higher in the ICH + vehicle group compared with the sham group, and it continued to increase after administration of KIRA6 (*p* < 0.05, Figure 5H).

We found that the body weight of mice slightly decreased in the ICH + vehicle group and the ICH + KIRA6 group compared with the Sham group 1 h after ICH, but the difference between the three groups was not statistically significant. However, 1 day after ICH, the body weight of mice in the ICH + vehicle group was significantly lower compared to the Sham group (*p* < 0.05, Figure 5I), and KIRA6 further reduced the body weight compared to the ICH + vehicle group (*p* < 0.05, Figure 5I).

**FIGURE 6:**
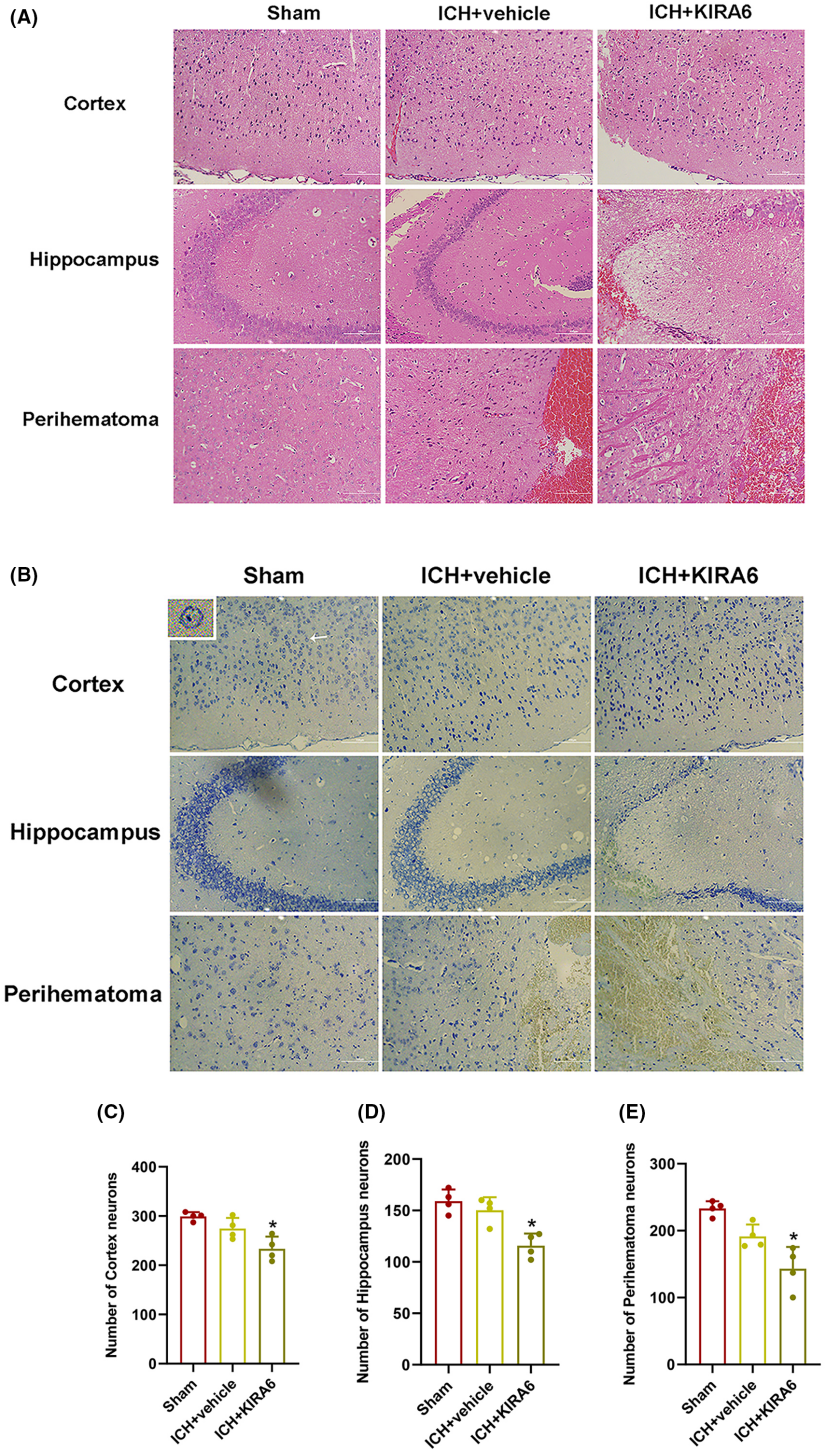
Effects of intranasal administration of KIRA6 on brain impairment after ICH. (A) Representative photomicrographs of each group of HE‐stained brain sections. (B) Representative photomicrographs of each group of Nissl‐stained brain sections. The heads of the arrows point to the surviving neurons. Scale bars = 100 μm. (C) Quantification of the number of surviving neurons in the cerebral cortex. (D) Quantification of the number of surviving neurons in the hippocampus. (E) Quantification of the number of surviving neurons in the perihematoma. Scale bars = 100 μm (*n* = 4). Data are the mean ± SD. *p < 0.05 vs. ICH + vehicle group.

To assess the pathological changes and neuronal damage in the brain tissue in ICH mice, HE staining and Nissl staining were performed. As shown in Figure 6A, HE staining of the brain tissue showed that neurons have clear nuclei and distinct nucleoli in the sham group. On the contrary, a part of neurons were slightly atrophy and degeneration at 12 h after ICH, and KIRA6 aggravated the pathological changes. Nissl staining of the brain tissue showed that there was a large number of neurons in the sham group, with clear nuclei and lightly staining. The number of surviving neurons at 12 h after ICH was slightly decreased compared with the sham group, but this difference was not statistically significant. However, the administration of KIRA6 significantly decreased the number of surviving neurons (*p* < 0.05, Figure 6B–E).

**FIGURE 7:**
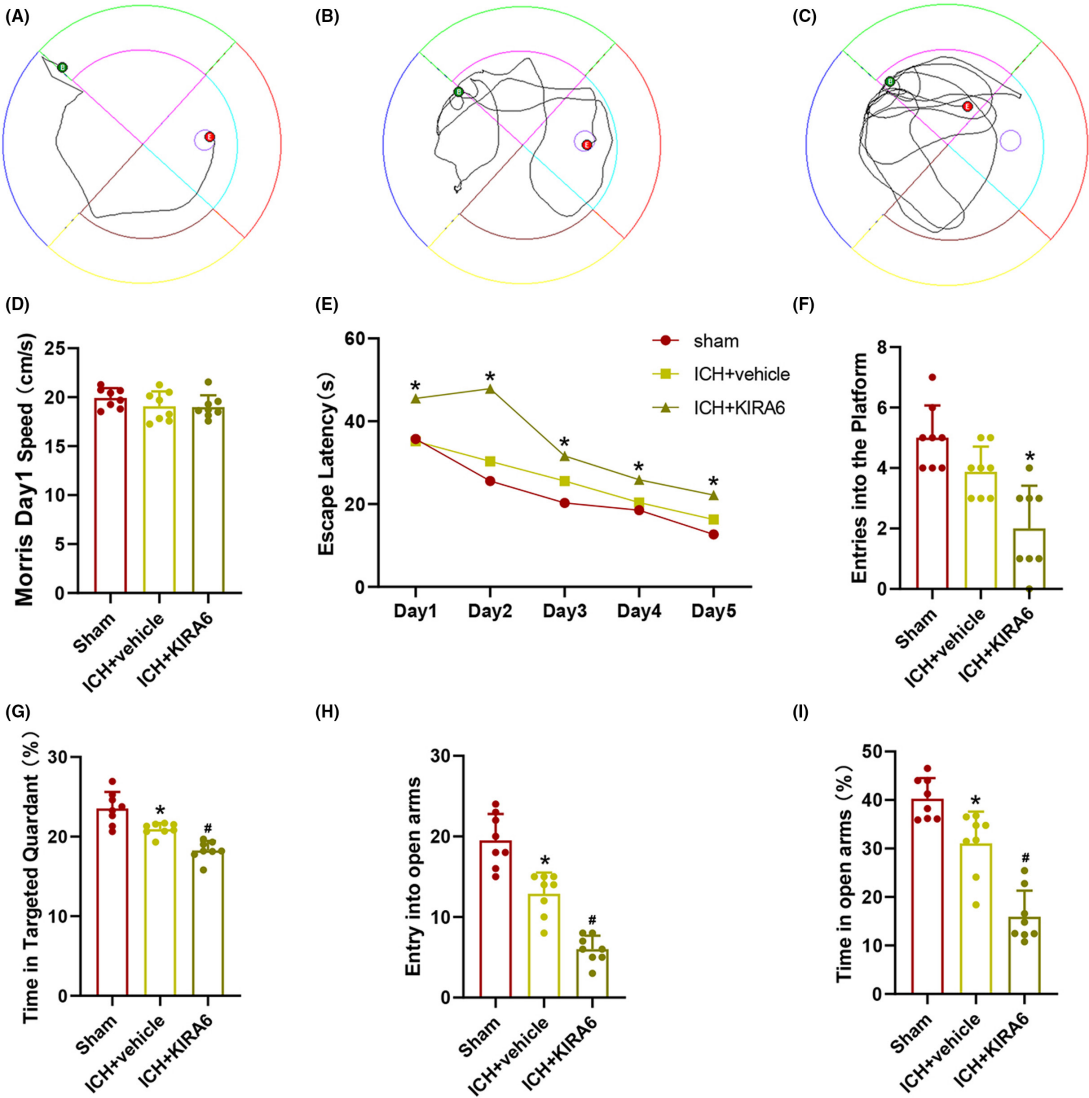
Effects of intranasal administration of KIRA6 on spatial learning and memory abilities and anxiety levels. (A–C) A representative swimming path diagram of each group at Day 5 (*n* = 8). The green color represents the starting point, and the red color represents the endpoint. (D) The Morris speed at Day 1 in the groups of Sham, ICH + vehicle, and ICH + KIRA6 (*n* = 8). (E) Escape latency to the platform from day one to day five of each group (*n* = 8). Values are presented as mean ± SD. *p < 0.05 vs. Sham group; ^#^ *p* < 0.05 vs. ICH + vehicle group. (F) Number of times entering the platform during the probe test (*n* = 8). Values are presented as mean ± SD. *p < 0.05 vs. ICH + vehicle. (G) Duration spent in the targeted quadrant during the probe test (*n* = 8). (H) Number of times entering the open arm (*n* = 8). (I) Percentage of time spent in open arms (*n* = 8). (G–I) Values are presented as mean ± SD. *p < 0.05 vs. Sham group; ^#^ *p* < 0.05 vs. ICH + vehicle group.

In order to assess the spatial learning ability, the Morris water maze test was used. During the spatial acquisition phase, as shown in Figure 7A–C, the swimming paths of the sham group, ICH + vehicle group and ICH + KIRA6 group on the fifth day were shown. The results showed no differences in spatial bias or basal swimming speed (Figure 7D). We also measured the escape latency, which is the time spent by the mice to find the hidden platform. The escape latency of the mice in all three groups decreased over the five consecutive training days, but the escape latency of the ICH + vehicle was still significantly higher compared to the sham group at Day 5, and ICH + KIRA6 group was also significantly higher compared to the ICH + vehicle group (Figure 7E, *p* < 0.05). In the probe test, when the platform was removed, the time spent in the platform zone decreased for each group compared to the sham group (*p* < 0.05), but the decrease in the ICH + vehicle group was not statistically significant (Figure 7F). Additionally, compared to the sham group and ICH + vehicle group, the ICH + KIRA6 group spent significantly less time in the target quadrant (*p* < 0.05, Figure 7G). These results indicate that KIRA6 exacerbates spatial learning and memory impairments in mice after cerebral hemorrhage.

In order to assess the impact of KIRA6 on anxiety levels in mice after cerebral hemorrhage, the elevated plus maze test was utilized. We recorded the number of entries into the open arms and the percentage of time spent in the open arms. A higher number of entries into the open arms or a longer duration of stay in the open arms indicates less severe anxiety in mice, whereas the opposite suggests more severe anxiety. Compared to the sham group, the ICH + vehicle group showed a decrease in both the number of entries into the open arms and the percentage of time spent in the open arms, which was further exacerbated after administration of KIRA6 (*p* < 0.05, Figure 7H–I).

### 3.6 Nox4 gene was a direct target gene of miR‐25‐3p

**FIGURE 8:**
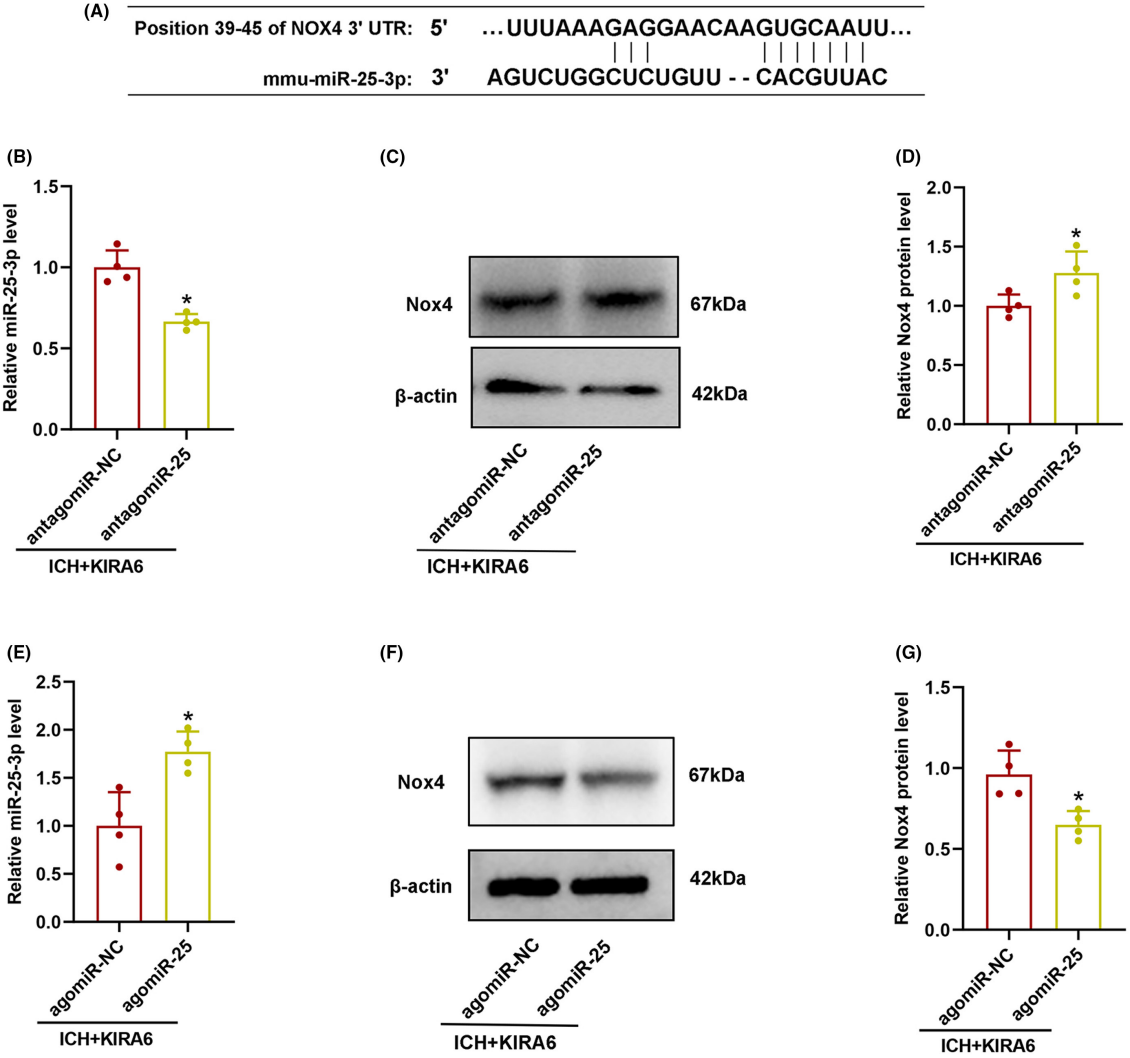
Effects of miR‐25‐3p antagomir or agomir on the expression of miR‐25‐3p and Nox4. (A) Sequence alignment showing putative miR‐25‐3p binding sites within the 3’‐UTR of the Nox4 mRNA in mice (http://www.mirdb.org/; https://www.targetscan.org/). (B) The miR‐25‐3p expression level was measured through RT‐qPCR (*n* = 4). (C) Representative western blot bands of Nox4 in the ICH + KIRA6 + antagomiR‐NC and ICH + KIRA6 + antagomiR‐25 groups. (D) Quantitative analysis of the relative expression level of Nox4 (*n* = 4). Values are presented as mean ± SD. *p < 0.05 vs. ICH + KIRA6 + antagomiR‐NC. (E) The expression levels of miR‐25‐3p were measured by RT‐qPCR (*n* = 4). (F) Representative western blot bands of Nox4 in ICH + KIRA6 + agomiR‐NC and ICH + KIRA6 + agomiR‐25 groups. (G) Quantitative analysis of the relative expression level of Nox4 (*n* = 4). Data are the mean ± SD. *p < 0.05 vs. ICH + KIRA6 + agomiR‐NC group.

To investigate whether Nox4 is a target gene of miR‐25‐3p, two online target prediction software, Targetscan and miRDB, were searched. It was found that miR‐25‐3p contains nucleotide sequences complementary to the 3′‐UTR sequence of Nox4 mRNA (Figure 8A).

### 3.7 The effect of miR‐25‐3p antagomir and agomir on Nox4 expression and oxidative stress in KIRA6‐administrated ICH mice

In order to confirm if miR‐25‐3p was involved in the regulatory role of IRE1α on Nox4 after ICH, miR‐25‐3p antagomir and agomir were used in KIRA6‐adminstrated ICH mice, and miR‐25‐3p and Nox4 expression were detected by RT‐qPCR and western blot, respectively.

RT‐qPCR data showed that antagomiR‐25 downregulated miR‐25‐3p expression and upregulated Nox4 expression (*p* < 0.05, Figure 8B–D), whereas agomiR‐25 upregulated miR‐25‐3p expression and downregulated Nox4 expression, and reversed the increase of Nox4 promoted by KIRA6 (*p* < 0.05, Figure 8E–G).

**FIGURE 9:**
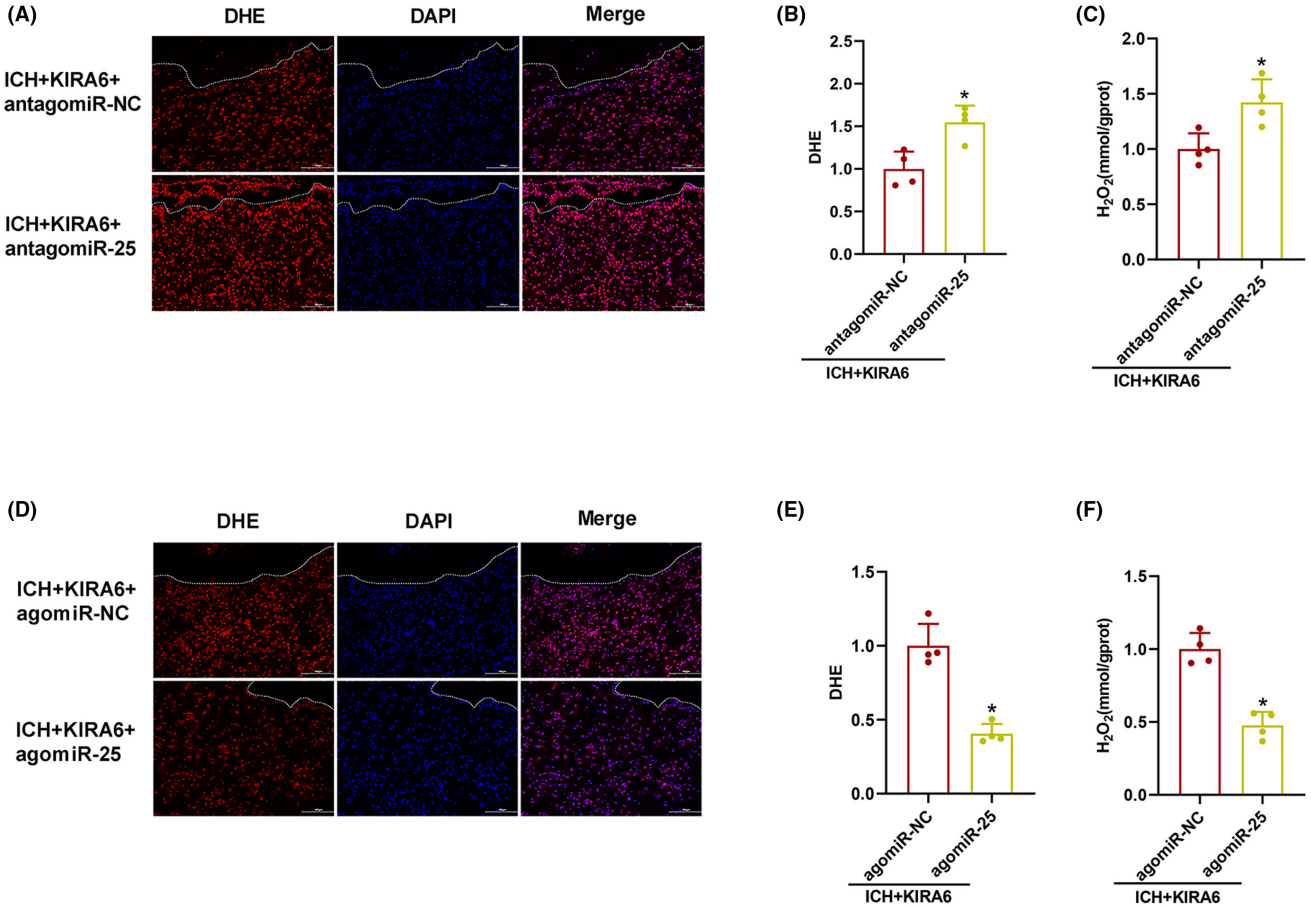
Effects of miR‐25‐3p antagomir or agomir on oxidative stress. (A) Representative DHE staining images around the hematoma in ICH + KIRA6 + antagomiR‐NC and ICH + KIRA6 + antagomiR‐25 groups. The boundary of the hematoma is indicated by the dotted line. Scale bar = 100 μm. (B) Quantification of the intensity DHE (red fluorescence) by Image J software (*n* = 4). (C) The measurement of H_2_O_2_ contents in ICH + KIRA6 + antagomiR‐NC and ICH + KIRA6 + antagomiR‐25 groups (*n* = 4). Values are presented as mean ± SD. *p < 0.05 vs. ICH + KIRA6 + antagomiR‐NC group. (D) Representative DHE staining images around the hematoma in ICH + KIRA6 + agomiR‐NC and ICH + KIRA6 + agomiR‐25 groups. The boundary of the hematoma is indicated by the dotted line. Scale bar = 100 μm. (E) Quantification of the intensity DHE (red fluorescence) by Image J software (*n* = 4). (F) The measurement of H_2_O_2_ contents in ICH + KIRA6 + agomiR‐NC and ICH + KIRA6 + agomiR‐25 groups (*n* = 4). Data are the mean ± SD. *p < 0.05 vs. ICH + KIRA6 + agomiR‐NC group.

Similarly, the intensity of DHE fluorescent and H_2_O_2_ contents were enhanced in ICH + KIRA6 + antagomiR‐25 compared with ICH + KIRA6 + antagomiR‐NC group (*p* < 0.05, Figure 9A–C), while agomiR‐25 decreased the intensity of DHE fluorescence and H_2_O_2_ contents (*p* < 0.05, Figure 9D–F).

### 3.8 The role of miR‐25‐3p antagomir and agomir on neurological function, brain edema, and hematoma volume in KIRA6‐administrated ICH mice

**FIGURE 10:**
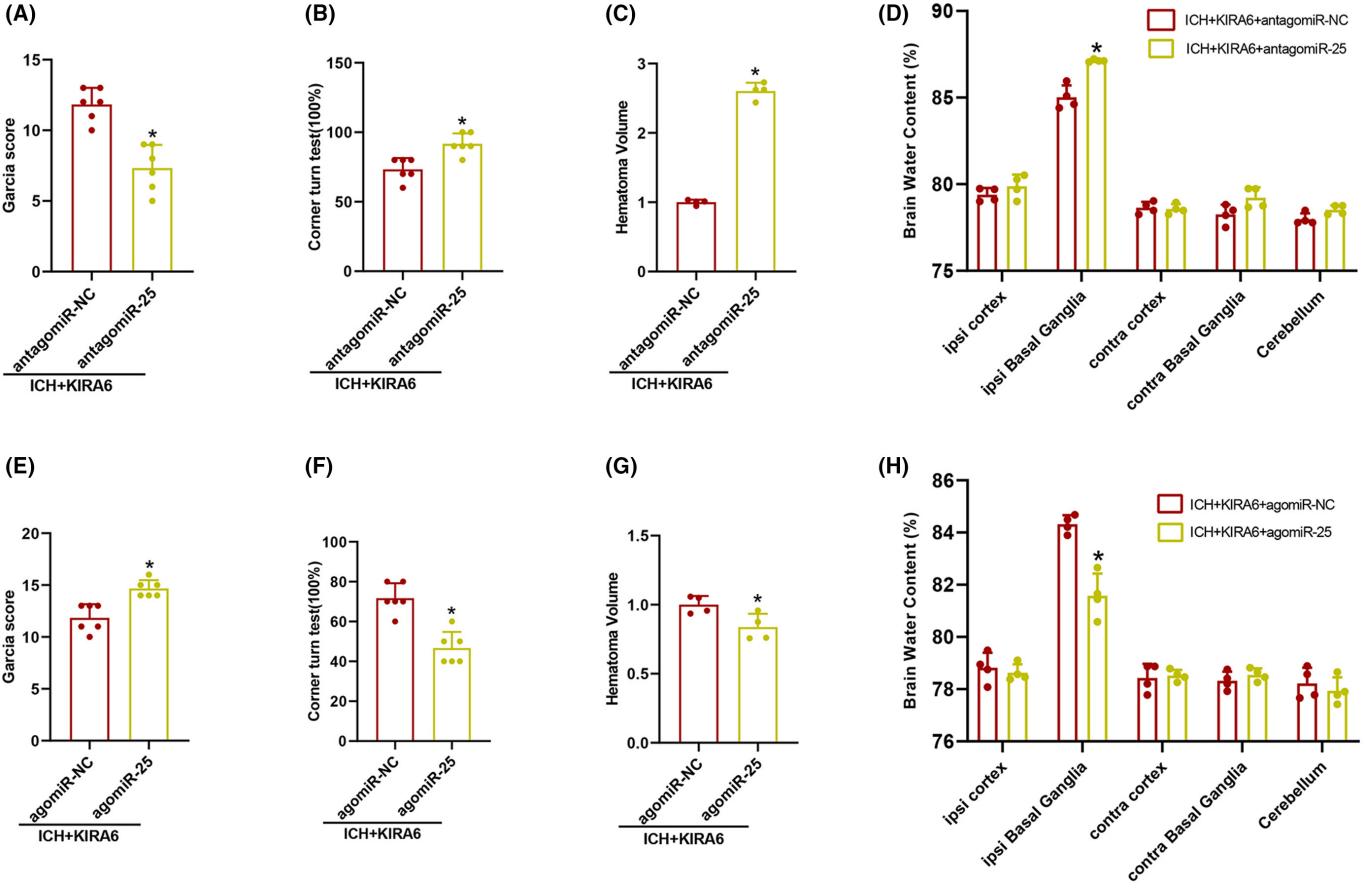
Effects of miR‐25‐3p antagomir or agomir on brain edema, hematoma volume, and neurological function. (A) The composite Garcia score were evaluated in the ICH + KIRA6 + antagomiR‐NC and ICH + KIRA6 + antagomiR‐25 groups (*n* = 4). (B) The corner turn times were evaluated in ICH + KIRA6 + antagomiR‐NC and ICH + KIRA6 + antagomiR‐25 groups (*n* = 4). (C) The analysis of hematoma volume in ICH + KIRA6 + antagomiR‐NC and ICH + KIRA6 + antagomiR‐25 groups (*n* = 4). (D) The analysis of brain edema in the ICH + KIRA6 + antagomiR‐NC and ICH + KIRA6 + antagomiR‐25 groups (*n* = 4). Values are presented as mean ± SD. *p < 0.05 vs. ICH + KIRA6 + antagomiR‐NC group. (E) The analysis of composite Garcia score in ICH + KIRA6 + agomiR‐NC and ICH + KIRA6 + agomiR‐25 groups (*n* = 4). (F) The analysis of corner turn times in ICH + KIRA6 + agomiR‐NC and ICH + KIRA6 + agomiR‐25 groups (*n* = 4). (G) The analysis of hematoma volume in ICH + KIRA6 + agomiR‐NC and ICH + KIRA6 + agomiR‐25 groups (*n* = 4). (H) The analysis of brain edema in the ICH + KIRA6 + agomiR‐NC and ICH + KIRA6 + agomiR‐25 groups (*n* = 4). Data are the mean ± SD. *p < 0.05 vs. ICH + KIRA6 + agomiR‐NC group.

Compared with the ICH + KIRA6 + antagomiR‐NC group, the Garcia score decreased in antagomiR‐25 (*p* < 0.05, Figure 10A). The number of right turns, hematoma volume, and brain water content increased in antagomiR‐25 compared with the ICH + KIRA6 + antagomiR‐NC group (*p* < 0.05, Figure 10B–D).

In contrast, agomiR‐25 increased the Garcia score, decreased the number of right turns, decreased hematoma volume and brain water content compared with the ICH + KIRA6 + agomiR‐NC group (*p* < 0.05, Figure 10E–H). These data suggested that miR‐25‐3p has a protective effect on neurological function after ICH.

## 4 DISCUSSION

IRE1α is an ER transmembrane protein, which contains an N‐terminal ER luminal domain responsible for stress sensing and C‐terminal kinase and endoribonuclease domain in the cytosol.^38^ The accumulation of unfolded/misfolded proteins triggers the inactive monomeric state IRE1α dimerization upon ER stress, which activates the kinase and IRE1α self‐phosphorylation. Then the self‐phosphorylation induces a conformational change and leads to the activation of IRE1α RNase activities, which take action on the excision of the mRNA encoding the transcription of X‐box binding protein‐1 (XBP1) or other multiple RNAs, including mRNAs, ribosomal RNAs, and microRNAs.^38^ The spatial and temporal expression patterns of IRE1α in ICH mouse brain were detected in the current study. The results showed that IRE1α is widely expressed on astrocytes, neurons, and microglia in various brain regions, which is consistent with other previous research results that IRE1α is expressed extensively in various mammalian cells.^38^ The time course results presented the ratio of p‐IRE1α/ IRE1α relative expression level increased in a time‐dependent manner, which indicated that the phosphokinase activity is activated in ICH mouse brain.

We initially speculated before the experiment that KIRA6, the inhibitor of IRE1α phosphokinase, can inhibit the RNase activity by inhibiting the IRE1α self‐phosphorylation and block the cleavage of miR‐25‐3p, so as to upregulate the expression level of miR‐25‐3p. However, our PCR results here showed that the expression level of miR‐25‐3p is decreased by KIRA6 instead of being upregulated. This result is conflicting to our expectations and suggests that the kinase and RNase activities of IRE1α are uncoupled completely, in other words, the downstream effects of kinase activation include RNase activation, but are not limited to RNase activation. The downregulation of miR‐25‐3p by kinase inhibitor KIRA6 in ICH mouse brain should be through a pathway unrelated to RNase activity blockage. In our previous experiments,^28^ the IRE1α RNase inhibitor STF083010 upregulated the expression of miRNA‐125‐b‐2‐3p and promoted the recovery of neurological function in ICH mice brain, which contradicts the results in the current study, that the IRE1α kinase inhibitor KIRA downregulated the expression of miRNA‐25‐3p and decreased the neurological function score of ICH model mice. The two kinds of IRE1α inhibitors, STF083010 and KIRA6, causing opposite outcomes in miRNAs expression may be due to that miRNA‐125‐b‐2‐3p and miRNA‐25‐3p are controlled by different kinase downstream pathways: miRNA‐125‐b‐2‐3p expression is mainly regulated by the RNase activity, so the inhibition of RNase activity by STF083010 will leads to the blockage of miRNAs cleavage and upregulated the expression of miRNA‐125‐b‐2‐3p as expected; while miRNA‐25‐3p is regulated principally by other kinase downstream phosphorylation pathway after kinase inhibitor KIRA6 administration and be downregulated. Which pathway(s) mediate(s) IRE1α kinase activity regulating on miR‐25‐3p expression needs to be further explored in future experiments.

Although increasingly studies have paid attention to the regulation role of miR‐25‐3p on the extent of oxidative stress,^24^, ^39^, ^40^ there is little research on the expression pattern of miR‐25‐3p following ICH and its relationship with oxidative injury in ICH brain. In this experiment, our results showed that the level of miR‐25‐3p was significantly increased following ICH injury. The fluorescence intensity of DHE staining and the content of H_2_O_2_ in ICH brain were increased by antagomiR‐25 and decreased by agomiR‐25. Accordingly, the neurological function score was reduced by antagomir‐25 and increased by agomiR‐25. The above results suggest that miR‐25‐3p can inhibit oxidative stress injury following ICH, and the upregulation of miR‐25‐3p in the early stage of ICH brain is a protective adaptive response.

ROS are a category of oxygen‐derived small molecules in the cell and include highly reactive free oxygen radicals such as superoxide anion (O_2_^−^) and stable nonradical oxidants such as H_2_O_2._ ^23^, ^41^ The brain is highly vulnerable to oxidative injury due to the high O_2_ consumption, and excessive ROS production during oxidative stress following ICH could induce brain injury through a variety of different mechanisms,^42^ including destroying membrane integrity by promoting lipid peroxidation, inducing autophagy mediated by activation of various autophagy‐related protein, leading to neuronal apoptosis by DNA and protein damage, initiating inflammatory response by activation of inflammasomes, and so on. Studies have shown that ER stress can affect the production of ROS through a series of complex ways.^43^, ^44^ For example, one of the ER stress markers, C/EBP homologous protein (CHOP), could induce the activation of endoplasmic reticulum oxidoreductase (ERO1α).^45^, ^46^ Then the activated ERO1α promotes the ROS production in ER by the transfer of electrons from the enzyme protein disulfide isomerase (PDI) to molecular oxygen,^43^, ^47^ and induces Ca^2+^ release by stimulating various receptors such as inositol‐1,4,5‐trisphosphate receptor (IP3R) or RyRs.^48^, ^49^ At last, the mitochondrial metabolism and production of ROS will be stimulated by the ROS produced from the ER and the released Ca^2+^.^50^, ^51^ Currently, the conclusion about whether ER stress promotes or inhibits ROS production in oxidative stress injury is controversial in numerous research. In addition to the results that CHOP promotes the production of ROS through a complex process in ER stress as seen in the above example, studies have also shown that PERK, an unfolding/misfolding protein sensor in ER stress, can phosphorylate Nrf2, and then Nrf2 dissociates from the Nrf2/KEAP1 complex and enters into the nucleus to promote antioxidant gene expression, leading to a reduction in ROS production.^52^, ^53^, ^54^ The contradictions in these conclusions may be in that some studies just focused on one or some specific pathways among those numerous pathways activated in ER stress, some of which play protective roles while some others aggravate oxidative stress. So the crosstalks between ER stress and oxidative stress after ICH are intricate and requires comprehensive and in‐depth research to shed light on it. It was observed in the present experiment that DHE fluorescence intensity and H_2_O_2_ contents were increased by KIRA6 administration, which demonstrated that the inhibition of IRE1α phosphokinase increased the contents of ROS. This result suggested that the activation of IRE1α phosphokinase during ER stress is responsible for downregulating the level of oxidative stress injury following ICH.

Nox enzyme family consist of seven members, Nox1, 2, 3, 4, 5 and Duox1, 2. Among them, Nox4 appears to produce mainly H_2_O_2_, which is different from that the mixture of free oxygen radicals and nonradical oxidants could be produced by Nox1‐3 and Nox5. Nox4 is extensively expressed in the central nervous system and growing evidences present that Nox4 acts as an important contributor to oxidative injury in pathophysiologic conditions such as ischemia, hemorrhage, and neurodegenrative processes.^23^, ^55^, ^56^, ^57^ Nox4 was discovered to be widely expressed in neurons, astrocytes, vascular endothelial cells, and microglia in the brains of rats after ICH, according to Xie et al.^56^ The level of ROS contents was significantly decreased, and the neurobehavioral score was increased after Nox4 knockdown. In our present study, the change pattern of Nox4 expression observed in ICH mouse model is consistent with Xie's former result in the rat model, which indicates that the increase of Nox4 expression is a common phenomenon in ICH brain.^56^ To explore whether IRE1α activation plays a regulating role on Nox4, we detected Nox4 expression under KIRA6 administration conditions and found that the expression level of Nox4 protein was upregulated after the phosphorylation level of IRE1α was inhibited by KIRA6. These results showed that the protective effect of IRE1α phosphokinase activation on oxidative stress injury after ICH can be achieved at least partly by regulating Nox4.

Since IRE1α phosphokinase can regulate the expression of both miR‐25‐3p and Nox4, whether there is a regulatory relationship between miR‐25‐3p and Nox4 is investigated in this study. Our results demonstrated that Nox4 was upregulated by antagomiR‐25 and downregulated by agomiR‐25, which verified the regulatory effect of miR‐25‐3p on Nox4. Fu et al^58^ pointed out that miR‐25 may regulate NOX4 gene expression through degradation and destabilization of Nox4 mRNA. They discovered that introducing miR‐25 precursor reduced endogenous Nox4 mRNA expression and decreased Nox4 mRNA t_1/2_, indicating that miRNAs can inhibit protein expression and cause mRNA degradation and destabilization. Our research results from miRNA target prediction software demonstrated that miR‐25‐3p contains nucleotide sequences complementary to the 3′‐UTR sequence of Nox4 mRNA, which means that miR‐25‐3p could combine with Nox4 mRNA and affect post‐transcriptional gene expression regulation of Nox4. So the current experimental and research results confirmed that Nox4 is a regulatory target of miR‐25‐3p

Thus, our results demonstrated that IRE1α kinase inhibition could regulate the expression of miR‐25‐3p, and Nox4 is a downstream molecular of miR‐25‐3p, while Nox4 is crucial for oxidative injury after ICH. So the oxidative stress response in ICH brain may be regulated by the IRE1α phosphokinase through miR‐25‐3p/Nox4 pathway.

There are some limitations in our current research. Firstly, although our results verified that miR‐25‐3p is regulated by IRE1ɑ kinase activity, the underlying mechanism is unclear. Some questions, such as which signaling proteins were phosphorylated and involved in the pathway connecting the IRE1ɑ kinase activity and miR‐25‐3p, are waiting to be unlocked. Secondly, only a single IRE1ɑ inhibitor was used in the present study, additional inhibitors or activators of IRE1ɑ should be put to use in the following experiments so as to validate the conclusions. Thirdly, the pathogenesis of ICH are influenced by multiple pathways and networks of genes and proteins, while here we only investigated the IRE1ɑ/miR‐25‐3p/Nox4 pathway. Massive other pathways involved in the ER stress and oxidative stress needs to be unveiled in the future work.

## 5 CONCLUSIONS

**FIGURE 11:**
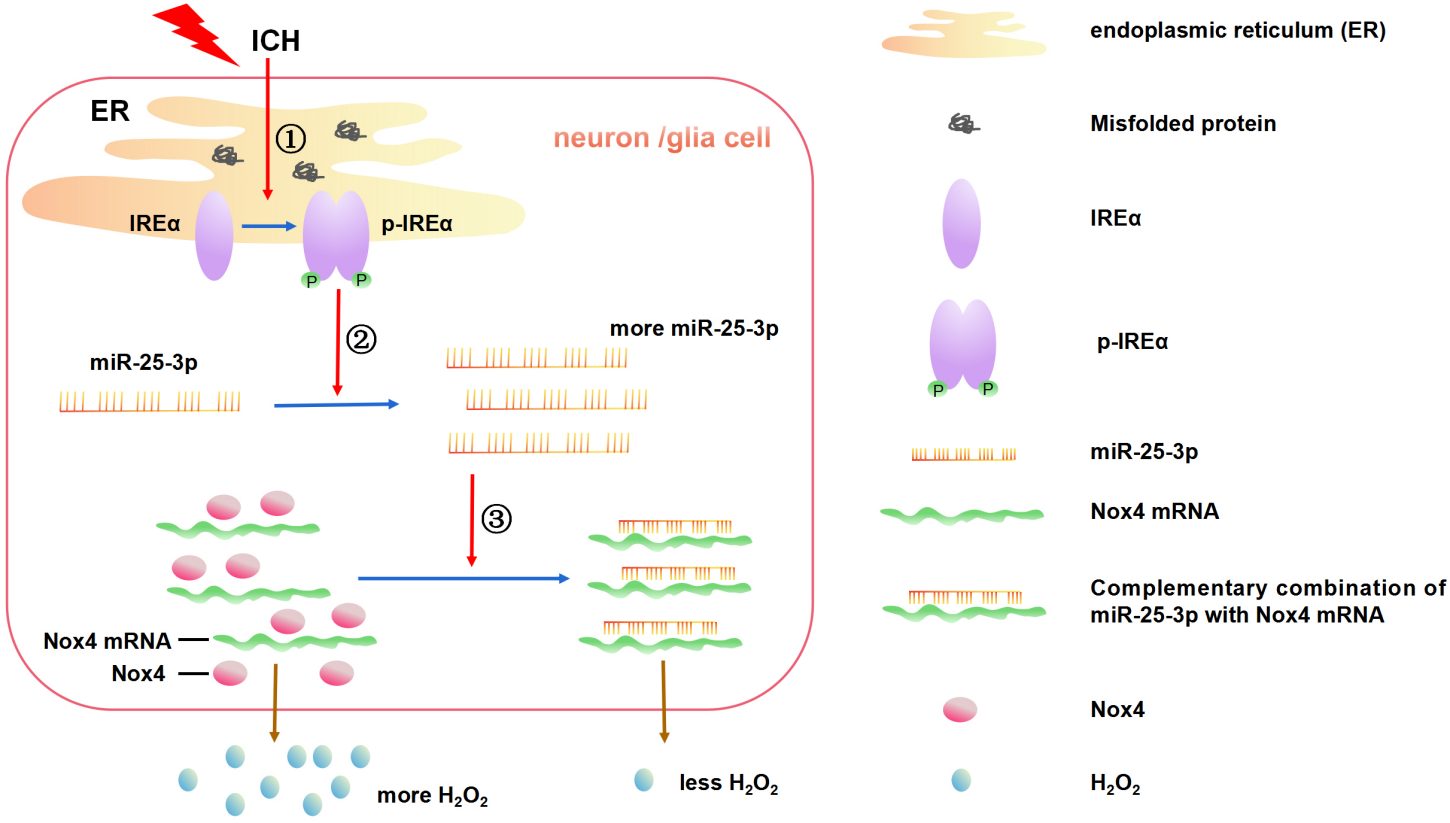
The proposed pathway by which IRE1α regulates the oxidative stress response via miR‐25‐3p/Nox4 in neuron and glia cells after ICH. ① ICH induces ER stress and activates the phosphokinase activity of IRE1α. ② Self‐phosphorylated IRE1ɑ increases the expression of miR‐25‐3p. ③ The complementary combination of miR‐25‐3p with Nox4 mRNA inhibits the translation of Nox4 mRNA and subsequently downregulated expression of Nox4, which leads to ROS production decrease, especially H_2_O_2_.

In conclusion, we demonstrated that inhibition of IRE1α phosphokinase downregulated the expression of miR‐25‐3p, upregulated the expression of Nox4 and ROS production, and exacerbated the oxidative injury following ICH. In addition, the expression of Nox4 and ROS production can be regulated by the intervention of miR‐25‐3p function. The oxidative stress response in ICH brain may be regulated at least partially by the IRE1α phosphokinase through miR‐25‐3p/Nox4 pathway in extensive neural tissue cells, including neurons, astrocytes, and microglia (Figure 11).

## FUNDING INFORMATION

This work was supported by the National Natural Science Foundation of China (NSFC82171457), Natural Science Foundation of Chongqing (CSTB2022NSCQ‐MSX1083), and Program for Youth Innovation in Future Medicine, Chongqing Medical University (W0031).

## CONFLICT OF INTEREST STATEMENT

The authors declare that they have no competing interests.

## Data Availability

The data are contained within the article.
